# Effects of skin and mild core cooling on cognitive function in cold air in men

**DOI:** 10.14814/phy2.15893

**Published:** 2023-12-19

**Authors:** Phillip J. Wallace, Dominique D. Gagnon, Geoffrey L. Hartley, Michael J. Taber, Stephen S. Cheung

**Affiliations:** ^1^ Environmental Ergonomics Laboratory, Department of Kinesiology Brock University St. Catharines Ontario Canada; ^2^ Faculty of Sports and Health Sciences University of Jyväskylä Jyväskylä Finland; ^3^ Clinic for Sports and Exercise Medicine, Department of Sports and Exercise Medicine, Faculty of Medicine University of Helsinki Mäkelänkatu Helsinki Finland; ^4^ School of Kinesiology and Health Sciences Laurentian University Sudbury Ontario Canada; ^5^ Department of Physical and Health Education Nipissing University North Bay Ontario Canada; ^6^ N^2^M Consulting Inc. St. Catharines Ontario Canada

**Keywords:** cognition, cold stress, core cooling, executive attention, skin cooling

## Abstract

This study tested the effects of skin and core cooling on cognitive function in 0°C cold air. Ten males completed a randomized, repeated measures study consisting of four environmental conditions: (i) 30 min of exposure to 22°C thermoneutral air (TN), (ii) 15 min to 0°C cold air which cooled skin temperature to ~27°C (CS), (iii) 0°C cold air exposure causing mild core cooling of ∆‐0.3°C from baseline (C‐0.3°C) and (iv) 0°C cold air exposure causing mild core cooling of ∆‐0.8°C from baseline (C‐0.8°C). Cognitive function (reaction time [ms] and errors made [#]) were tested using a simple reaction test, a two–six item working memory capacity task, and vertical flanker task to assess executive function. There were no condition effects (all *p* > 0.05) for number of errors made on any task. There were no significant differences in reaction time relative to TN for the vertical flanker and item working memory capacity task. However, simple reaction time was slower in C‐0.3°C (297 ± 33 ms) and C‐0.8°C (296 ± 41 ms) compared to CS (267 ± 26 ms) but not TN (274 ± 38). Despite small changes in simple reaction time (~30 ms), executive function and working memory was maintained in 0°C cold air with up to ∆‐0.8°C reduction in core temperature.

## 1 INTRODUCTION

Occupational workers, military personnel, and athletes often perform in cold environments, where maintaining cognitive and physical function is critical to preventing accidents, preserving operational capacities, and minimizing further thermal strain (Castellani & Tipton, 2016; Palinkas, 2001; Pilcher et al., 2002; Taber et al., 2016). Maintaining cognitive function is more demanding in cold compared to thermoneutral environments (~22°C) due to strong peripheral vasoconstriction reducing cerebral and muscle blood flow and oxygenation (Ferguson et al., 2018; Gibbons et al., 2020; Hodges et al., 2019), altered energy metabolism in part due to shivering if present (Haman et al., 2016), and decreased manual function and coordination due to cooled muscles and joints (Castellani et al., 2018; Cheung et al., 2007). Furthermore, psychological strain increases, with higher thermal discomfort (Hoffman, 2001) and alterations in mood (Muller et al., 2011). Collectively, these changes can lead to decrements in cognitive task performance in ambient air ≤10°C (Falla et al., 2021; Hancock et al., 2007; Pilcher et al., 2002). Specific decrements have been reported to include impaired executive function (Racinais et al., 2017), working memory (Shurtleff et al., 1994; Thomas et al., 1989), attention/vigilance (Sun et al., 2022; Watkins et al., 2014), and psychomotor processing (Teichner, 1958) with acute (30–120 min) passive cold air (range: −15 to 10°C) exposure.

Impaired cognitive performance in the cold is not a universal finding, as no effects on executive function (Makinen et al., 2006; Muller et al., 2012; Yang et al., 2021), working memory (Færevik et al., 2021; Makinen et al., 2006; Muller et al., 2012), attention/vigilance (Makinen et al., 2006), or psychomotor processing (Færevik et al., 2021; Makinen et al., 2006) have also been reported with cold air exposure. Differences in performance outcomes may be due to a variety of factors including the speed of cooling, intensity of cold environment, duration of exposure, and the cognitive test being performed (Falla et al., 2021; Hancock et al., 2007; Pilcher et al., 2002). However, a majority of the studies in cold air primarily reduce mean skin temperature rather than core temperature (Falla et al., 2021). This raises a fundamental question of whether significant or sufficient deep core body cooling was achieved, leaving largely unanswered the question of whether core cooling impairs cognitive function in cold air. Cooling skin temperature alone (without reductions in core temperature) causes vasoconstriction, mild shivering, and thermal discomfort (Shurtleff et al., 1994). Under these conditions, cognition is proposed to be impaired due to increased distraction and decreased arousal from thermal discomfort leading to fewer attentional resources available to complete cognitive tasks (Teichner, 1958). Whereas whole‐body cooling sufficient to induce mild hypothermia (decrease in core temperature by 0.5–2.0°C) further increases cold strain (increased shivering, thermal discomfort, heart rate, vasoconstriction) (Bittel et al., 1988; Hodges et al., 2019), and the lower core temperature may decrease brain temperature and increase neural strain in areas such as the prefrontal cortex, and subsequently influence cognitive function (Gibbons et al., 2020; Jones et al., 2019).

Based on previous findings, it could be reasonably suggested that there may be a core temperature threshold for impairment in cognitive function in cold air. For example, Ellis (1982) determined that 150 min of −12°C cold air exposure—leading to a ∆−0.8°C reduction in core temperature—impaired speed and accuracy on a serial choice reaction test (mathematical discrimination task) but did not impair executive function (Stroop test) or verbal reasoning. These impairments first started to occur within the first 30 min as skin temperature decreased, without changes in core temperature (Ellis, 1982). However, Taber et al. (2016) found no impairment to executive attention, executive function, working memory, psychomotor processing, or mental rotation throughout 24 h of cold exposure (7.5°C air) despite a sustained ~∆‐0.5°C reduction in core temperature. Similarly, Mäkinen et al. (2006) found no impairments in executive function, working memory, or psychomotor processing following a single exposure of 100 min of cold air exposure (10°C) leading to ~∆‐0.4°C in core temperature. Collectively, this limited data may indicate a potential core temperature threshold for impairment in cognitive function in cold air (Pilcher et al., 2002). However, a key limitation of these studies is that cold exposure was based on time, which fails to acknowledge the large individual responses and thus variations in cold strain among participants (Castellani & Tipton, 2016). In order to tease out if there is a cold air exposure‐response on cognitive function, we manipulated thermal strain based on changes in core temperature to incorporate individual differences in thermoregulatory capacity and normalized cold strain between participants (Cheung et al., 2007). Furthermore, we tested multiple levels of core temperature cooling to determine whether there is a threshold for cognitive task impairment.

The purpose of this study was to investigate a dose response to cold air exposure—ranging from skin/peripheral cooling through to two levels of core temperature decrease—on cognitive performance using executive function, working memory, and psychomotor processing tasks. To achieve these thermal states, we tested cognitive function in four distinct randomized conditions: thermoneutral (TN, 22°C); cold skin (CS) where skin temperature was lowered but not core temperature in cold air (0°C); and cold air exposures where core temperature decreased by either ∆‐0.3°C (C‐0.3°C) or ∆‐0.8°C (C‐0.8°C). We hypothesize that: (i) cognitive performance (speed and accuracy) will be impaired with CS compared to thermoneutral conditions, and (ii) cognitive performance will be further impaired with progressively greater levels of core cooling.

## 2 METHODS

### 2.1 Participants

The experimental protocol was cleared by the Research Ethics Board at Brock University (REB# 19‐026), conformed to the latest revision of the Declaration of Helsinki and was a part of a doctoral thesis (Wallace, 2023). Ten healthy male volunteers (age: 27.0 ± 9.8 years, mass: 77.9 ± 10.6 kg, height: 178.6 ± 3.7 cm, body fat: 13.3 ± 5.0%, body surface area: 1.93 ± 0.12 m^2^), who were free from cardiovascular, respiratory, neurological, and cold disorders (i.e., Raynaud's disease or cold urticaria) were recruited from the university and community population. All participants were informed of the experimental protocol and associated risks before participating in this experiment and provided both verbal and informed written consent. This study was limited to male participants and women were excluded due to fluctuations in resting core temperature across the menstrual cycle (Greenfield et al., 2021). There is potential for the menstrual cycle to influence cutaneous vasoconstriction, shivering, and non‐shivering thermogenesis (Greenfield et al., 2021) leading to potential sex‐related differences in cooling times. We did not test in a single phase to control for hormonal fluctuations as testing would be separated by 1 month (4 months to collect) and we did not know if cognitive performance would be maintained over a long testing window. It is currently unknown whether there are sex differences in cognitive function under cold stress.

### 2.2 Experimental design

The experiment was a randomized repeated measures design consisting of two familiarization sessions and four experimental sessions. The first familiarization session involved collecting anthropometric measures and practicing the cognitive test battery (CTB). The second familiarization was designed to reduce the potential of a learning effect through two further complete practices of the CTB. The four experimental conditions were in a randomized order separated by 3–7 days to minimize the potential of cold acclimation and performed at the same time of day to control for circadian fluctuations in core temperature. Participants were instructed to avoid vigorous exercise and alcohol consumption 24 h and caffeine 6 h prior to each experimental session.

### 2.3 Familiarization trials

Upon arrival for the first familiarization trial, anthropometric measurements (height (cm), mass (kg)), body surface area (m^2^) (Du Bois & Du Bois, 1916), and % body fat was calculated using the 7‐site skinfold technique (Jackson & Pollock, 1978). Participants then performed a familiarization of the CTB (see description below) in a thermoneutral environment (22°C). Upon arrival for the second familiarization trial, participants practiced the CTB twice more (separated by ~45 min) for a total of three times (Wallace et al., 2017). Familiarization was performed on multiple days as the selected CTB has demonstrated to have better familiarization when memory consolidation is allowed to occur (Jones et al., 2016). We compared performance from Familiarization 3 to TN for all cognitive variables using a paired samples *t*‐test and found no significant difference in reaction time or errors made (all *p* > 0.05). During all practice trials, participants wore winter gloves and a soft‐silicone mask that were identical to equipment used during the experimental trials.

### 2.4 Experimental trials

Upon arrival, participants voided their bladder and nude body mass (kg) was recorded. A urine sample was tested for urine specific gravity (PAL‐10S, Atago, Japan) to determine hydration status. Participants were considered euhydrated if USG was ≤1.020, or else the test was rescheduled (no trials were ultimately rescheduled from hypohydration). Participants were then instrumented (see below), entered an environmental chamber, and were seated on a chair and were provided with ear plugs. Participants then completed a 5‐min baseline sitting quietly with eyes closed in thermoneutral conditions (~22.0°C, ~50% relative humidity). Next, participants completed testing in one of the following four experimental conditions:

**Thermoneutral (TN)**—Participants remained seated in the chamber for 25 min (30‐min total) before being fitted with the winter gloves prior to commencing the CTB in the chamber.

**Cold Skin (CS)**—Participants remained seated in the environmental chamber as the ambient temperature was incrementally decreased to 0°C (~15–18 min) and wind speed was increased to 0.8–1.2 m/s using a fan. Once the chamber temperature reached 0°C, the fan was turned off and participants performed the CTB. This design allowed for the core temperature to remain neutral while skin temperature was reduced to approximately 27°C. This level of skin temperature change was used as the vasoconstrictory response is maximal at a mean skin temperature of 29.5–30°C (Greaney et al., 2015) and thus would lead to increased thermal discomfort.

**C**‐**0.3°C**—Participants remained seated in the environmental chamber as ambient temperature was decreased to 0°C and wind speed was increased to 0.8–1.2 m/s until their rectal core temperature (T_re_) dropped by ∆0.3°C. At this point the fan was turned off and participants performed the CTB.

**C**‐**0.8°C**—Participants remained seated in the environmental chamber as ambient temperature was decreased to 0°C and wind speed was increased to 0.8–1.2 m/s until the participants' T_re_ dropped by ∆0.8°C, whereupon the fan was turned off and participants performed the CTB.

For all cold trials, participants remained in the chamber and performed the CTB in cold air (0°C) which allowed for further cooling and continuous shivering to occur. For all cold trials, there was an institutional ethical cutoff of core temperature ≤35.0°C and an exposure limit of 150 min following chamber air temperature reaching 0°C. Three participants (30%) did not reach the desired ∆‐0.8°C *T*_re_ within the 150 min cutoff limit. Each of these participants started the CTB following the cutoff time with a ∆‐0.7°C *T*_re_ There were no observable differences in performance between these individuals and the rest of the participants.

### 2.5 Clothing

During TN trials, participants wore a cotton t‐shirt or cycling jersey, cycling bib shorts, socks, and athletic shoes (~0.26 clo ensemble) and were provided with winter gloves before commencing the CTB. In all three cold trials, participants wore the same clothing as TN plus a pair of track pants throughout the experimental trial. Following baseline, participants were fitted with earmuffs, gloves, and a fleece blanket around their shoes (~0.63 clo). Based on pilot testing, this additional equipment was deemed necessary to offset extreme discomfort of the extremities during cooling and minimize the risk of participant dropout.

### 2.6 Physiological measurements

Prior to baseline, participants were instrumented with a flexible thermocouple (RET‐1, Physitemp Instruments, USA), self‐inserted 15 cm beyond the anal sphincter to measure *T*_re_ (°C). Weighted mean skin temperature (${\bar{T}}_{\text{skin}}$, °C) was measured using thermistors (Concept Engineering, Old Saybrook, USA) collected at seven sites (Hardy et al., 1938):

$$\begin{array}{c} {\bar{T}}_{\text{skin}}=0.07_{\text{forehead}}+0.14_{\text{forearm}}+0.05_{\text{hand}}+0.35_{\text{abdomen}}\\ \;+0.19_{\text{thigh}}+0.13_{\text{shin}}+0.07_{\text{foot}} \end{array}$$

Forearm temperature (*T*_forearm_, °C) and hand temperature (*T*_hand_, °C) were analyzed to quantify the local cooling response as these sites were likely to influence the ability to respond during the CTB. Heart rate was calculated using R‐R intervals using a standard three‐lead electrocardiogram (MLA2340, AD Instruments; USA). Participants were fitted with a soft silicone facemask (7450V2, Hans Rudolph, USA) connected to an inline gas collection system (ML206 Gas Analyzer, AD Instruments; USA) calibrated following the manufacturer's instruction using air tanks containing 16% oxygen and 5% carbon dioxide. Expired gases were collected to continuously measure oxygen consumption ($\dot{V}O_{2}$, L.min^−1^), carbon dioxide expiration ($\dot{V}{\mathrm{CO}}_{2}$, L.min^−1^), respiratory exchange ratio (RER, $\dot{V}{\mathrm{CO}}_{2}/\dot{V}O_{2}$) to assess metabolic heat production ($\dot{M}$) which is primarily derived from shivering thermogenesis. If RER was <1.00 the following equation (Cramer & Jay, 2019) normalized to body surface area ($A_{D}$) was used to calculate $\dot{M}$:

$$\begin{array}{c} \dot{M}=\left(\dot{V}O_{2}∙\frac{\left[\left(\left(\frac{\mathrm{RER}-0.7}{0.3}\right)∙21.13\right)+\left(\left(\frac{1.0-\mathrm{RER}}{0.3}\right)∙19.62\right)\right]\;}{60}\times 1000\right)\\ \;/A_{D}\;\left[W∙m^{2}\right] \end{array}$$

If RER ≥1, the following equation was used to account for the energy equivalent for carbohydrates only (Cramer & Jay, 2019):

$$\dot{M}\;\left(\mathrm{RER}\geq 1.0\right)=\left(\dot{V}O_{2}∙\frac{21.13\;}{60}\times 1000\right)/A_{D}\;\left[W∙m^{2}\right]$$

All physiological data were averaged over the 5‐min baseline and while performing the tasks during the CTB (rest in between tasks and practice was not included).

### 2.7 Perceptual measures

Subjective assessments of the environmental conditions were assessed using a 1–4 scale to measure thermal comfort and a 1–7 scale for thermal sensation (Gagge et al., 1967) and a 0–10 scale (0 = rest, 3 = moderate, 5 = hard, 7 = very hard, 10 = maximal effort), which was modified from a 0 to 10 Borg scale, was used to measure perceived mental exertion of the cognitive tests and were collected upon the completion of the CTB.

### 2.8 Cognitive test battery

To measure progressive changes in cognitive function, participants performed an ~15‐min CTB using the Dalhousie Cognitive Assessment Battery (DalCAB) (Jones et al., 2015, 2016). The DalCAB is a validated assessment tool to measure executive attention (Jones et al., 2015, 2016) and is susceptible to impairment in learning with sleep deprivation (Cunningham et al., 2018). The chosen tasks consisted of a simple reaction time task, vertical flanker task, and item working memory task. These tasks were selected as they have been shown to measure a part of the executive control of attention referred to as executive attention, which is comprised of several different cognitive processes including executive function, working memory, attention, and vigilance and share similar neural structures and pathways within the executive attention network (Jones et al., 2015, 2016; McCabe et al., 2010). The most common similarities between the cognitive functions is attentional ability, maintaining a goal in an active state during a task and to resolve interference and filter out distractions (McCabe et al., 2010). Due to the shared nature of these cognitive processes, we aimed to test multiple executive attention functions (e.g., working memory, attention, filtering, executive function) and simple task performance (e.g., psychomotor processing speed) to determine if task‐dependent changes in cognition occur in cold air exposure.

To ensure similar manual dexterity requirements between trials, participants wore winter gloves for all cognitive testing. Furthermore, in pilot testing, it was determined that the glove thickness caused difficulty responding (using keyboard keys) causing false misses and errors. To minimize these errors, we affixed an analogue thumbstick (1 cm diameter) to the “caps lock” and “enter” keys creating a raised platform (2.5 cm in height) for easier responding and minimizing the manual dexterity required to respond (i.e., multiple fingers could be used to respond if needed). For all tests, the reaction time (RT) was averaged only using correct trials.

### 2.9 Simple reaction time task

The simple reaction time task assessed psychomotor processing function and vigilance. For this test, a turned playing card (French deck) was presented in the middle of the screen and the participant was asked to respond as soon as the card flipped over. Participants used their dominant hand to respond. A total of 60 stimuli were presented with a maximal response time of 1000 ms. Response–stimulus intervals (RSI) were randomly set at 500, 1000, and 1500 ms to minimize anticipatory responses. Furthermore, the varied response–stimulus intervals provide an index of vigilance through a temporal preparation effect, where healthy individuals respond faster when given a longer RSI (i.e., 1500 ms) compared with a shorter RSI (i.e., 500 ms) due to a longer preparation time (Jones et al., 2015, 2016). Performance was measured as RT (ms) and accuracy (%) on all trials. Reaction time was also quantified for each RSI. Furthermore, a preparation effect was calculated as the difference between 1500 and 500 ms RSI.

### 2.10 Vertical flanker task

The flanker task is used as a measure of executive function based on selective attention, filtering, and/or conflict resolution. In this task, a central target stimulus is presented with two flanking stimuli (flankers) above and below that are either the same as (congruent) or different than (incongruent) the central target stimulus. The participant had to decide and respond regarding a feature of the central stimulus (e.g., red heart or red diamond) while ignoring/filtering the flanking stimuli. This creates a flanker effect where participants respond faster with fewer errors on congruent compared to incongruent stimuli (Jones et al., 2015, 2016). The array was slightly offset vertically for each stimulus display in order to reduce attentionally spotlighting on the central stimulus, while also allowing flankers to remain visible throughout the task. A total of 100 stimuli were presented with a maximal response time of 1500 ms. The variables measured were the RT (ms), number of errors, and accuracy on congruent, incongruent, and total trials. Furthermore, an interference effect was calculated as difference in response times between incongruent and congruent stimuli.

### 2.11 Item working memory task

The item working memory task (identity Sternberg task) is a measure of working memory capacity where participants are presented with a series of memory sets of stimuli to be measured. The stimulus set is followed after a delay by a single probe stimulus. There were three set sizes (two, four, six items) where participants were presented with a series of non‐repeating stimuli (playing cards) and had to respond if the probe stimulus was present or absent in the previously viewed stimulus set. Set presentations were randomized where a total of 30 series were presented (10 of each set size) with a maximum RT allowed of 3000 ms. In healthy individuals, as the number of items in the set increases, the number of errors increase, and the RT required to decide about the probe stimulus also increases (Jones et al., 2016). The variables measured were RT (ms), number of errors, and accuracy (%) for the two, four, and six item and total sets.

### 2.12 Statistical analysis

All physiological and cognitive data are presented as mean ± SD. Physiological variables were assessed using a four condition (TN vs. CS vs. C‐0.3°C vs. C‐0.8°C) × 2 time (Baseline vs. CTB) repeated measures ANOVA. Simple reaction time task RT was assessed with a three RSI (500 vs. 1000 vs. 1500) × 4 condition repeated measures ANOVA. Vertical flanker RT, errors, and accuracy were assessed using a 2 flanker type (congruent vs incongruent) × 4 condition repeated measures ANOVA. Item working memory RT, errors, and accuracy were assessed with a 3 set size (two items vs. four items vs. six items) × 4 condition repeated measures ANOVA. Furthermore, the preparation effect of the simple reaction time task, accuracy for the simple reaction time task, and interference effect from the vertical flanker task were assessed using a 1 × 4 (condition) repeated measures ANOVA. Data were normally distributed and were also assessed by the Kolmogorov–Smirnov test. If sphericity was violated (*p* < 0.05), the Greenhouse–Geisser correction was used. A Bonferroni post hoc analysis corrected for multiple comparisons were used to test for specific main effects between task sets (e.g., RSI), conditions or between conditions and time. Significance was assumed with a *p* < 0.05.

All perceptual responses (ordinal data) are presented as median (quartile 1–quartile 3). Perceptual data were assessed using a 1 × 4 (condition) Friedman's ANOVA with a Wilcoxon‐Signed Rank test for post hoc analysis to compare between conditions. To reduce the likelihood of Type 1 error due to multiple comparisons, α value was revised based on number of comparisons (Ferguson et al., 2018), therefore *p* = 0.008. All statistical analyses were performed using SPSS statistics for Windows (SPPS Statistics for Windows, version 28; IBM Corp. USA).

## 3 RESULTS

### 3.1 Experimental design

**FIGURE 1:**
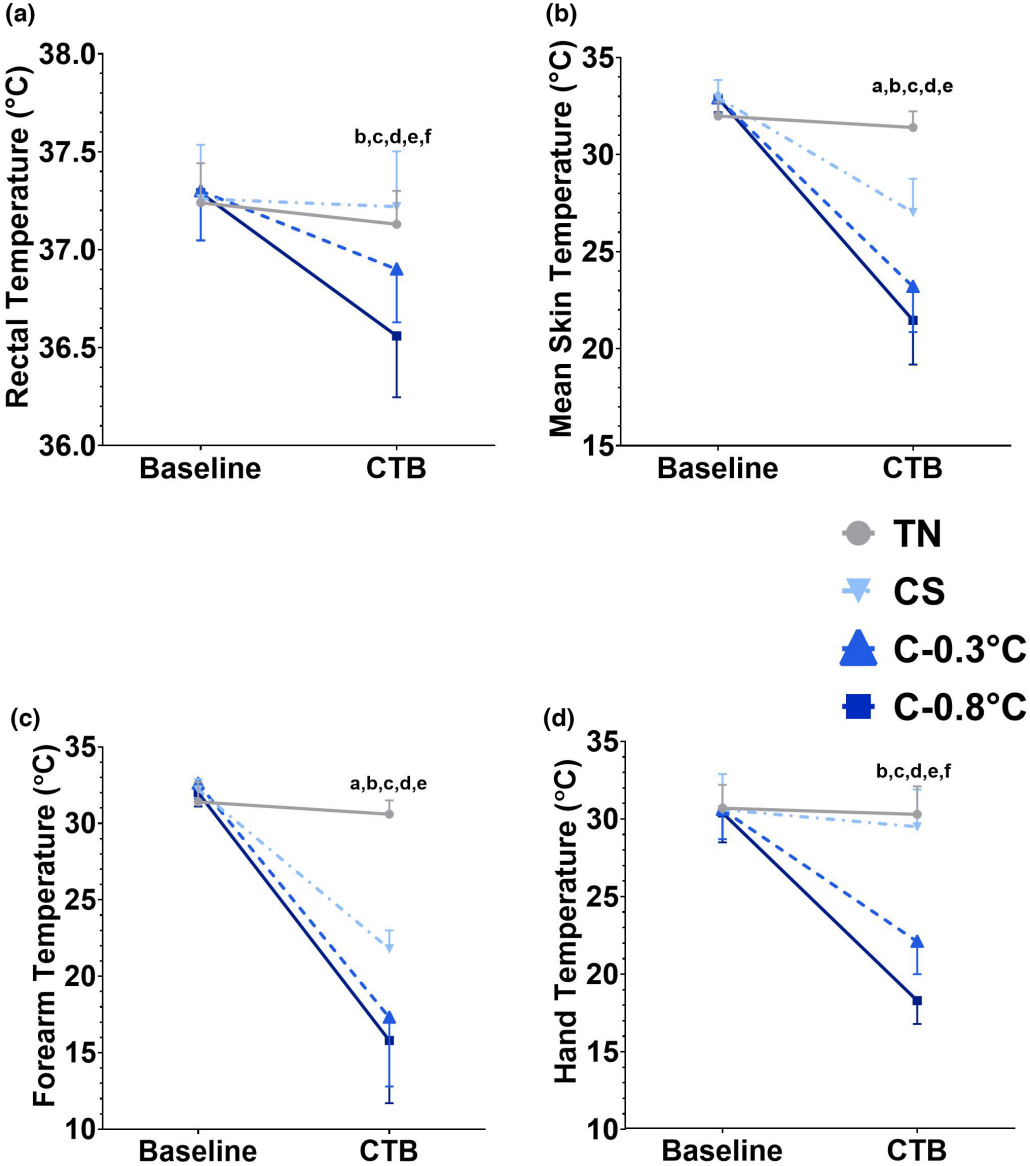
Thermal responses for core rectal temperature (panel A), whole‐body mean skin temperature (panel B), forearm temperature (panel C), and hand temperature (panel D) (*n* = 10 males). Data were analyzed using a 2 Time × 4 Condition repeated measure ANOVA. All data demonstrated a condition, time, and interaction effect (all *p* < 0.05). Pairwise comparisons can be interpreted as a = difference between TN and CS, b = difference between TN and C‐0.3°C, c = difference between TN and C‐0.8°C, d = difference between CS and C‐0.3°C, e = difference between CS and C‐0.8°C, f = difference between C‐0.3°C and C‐0.8°C. TN = thermoneutral, CS = Cold Skin/Shell, HYPO‐0.5°C = mild core cooling (hypothermia) of ∆‐0.5°C from baseline, HYPO‐1.0°C = mild core cooling (hypothermia) of ∆‐1.0°C from baseline.

Cooling times before commencing the CTB were CS: 19.0 ± 2.3 min, C‐0.3°C: 103.0 ± 37.2 min (range: 20–146 min), C‐0.8°C: 149.3 ± 32.2 min (range: 89–173 min). We were successful in creating four distinct experimental conditions. There was a condition, time, and interaction effect (all *p* ≤ 0.001) for *T*_re_ (Figure 1a), ${\bar{T}}_{\text{skin}}$ (Figure 1b), *T*_forearm_ (Figure 1c), and *T*_hand_ (Figure 1d), where pairwise comparisons determined no differences at Baseline between any of the conditions. By design, during the CTB, *T*_re_ was different between all conditions (all *p* ≤ 0.016) except TN versus CS (*p* = 0.667). Mean skin temperature was different between all conditions during the CTB except C‐0.3°C and C‐0.8°C (*p* = 0.132). Forearm temperature was lower in all three cooling conditions compared to TN (all *p* < 0.001) during the CTB. Furthermore, *T*_forearm_ was lower in C‐0.3°C (*p* = 0.040) and C‐0.8°C (*p* = 0.006) compared to CS. There were no differences for *T*_forearm_ between C‐0.3°C and C‐0.8°C (*p* = 0.811) during the CTB. Whereas, during the CTB, *T*_hand_ was different between all conditions (all *p* ≤ 0.005), except there were no differences between TN compared to CS (*p* = 0.945).

### 3.2 Perceptual responses

Perceptual measures are presented in Table 1 where all variables demonstrated a condition effect (all *p* ≤ 0.001). Overall, there was no difference in thermal comfort between TN and CS (*p* = 0.018) with significantly greater discomfort in C‐0.3°C and C‐0.8°C (both *p* = 0.004) relative to TN. Both C‐0.3°C (*p* = 0.004) and C‐0.8°C (*p* = 0.007) had greater discomfort compared to CS, with no difference between the two conditions (*p* = 0.317). Thermal sensation was perceived as significantly cooler relative to TN in all cold conditions (all *p* ≤ 0.007). There were no differences between the 3 cold conditions (all *p* ≥ 0.015). Despite condition effect for perceived mental exertion, there were no pairwise differences between the conditions in the post hoc analysis (all *p* ≥ 0.010).

**TABLE 1:** Perceptual responses collected following completion of the CTB presented as median (Quartile 1–Quartile 3) for the four experimental conditions (*n* = 10 males).

Variable	TN	CS	C‐0.3°C	C‐0.8°C
Thermal comfort (1–4)*	1 (1‐2)^cd^	3 (2–3.25)^cd^	4 (3‐4)^ab^	4 (4‐4)^ab^
Thermal sensation (1–7)*	4 (3‐4)^acd^	2 (1‐3)^a^	1 (1‐1)^a^	1 (1‐1)^a^
Mental exertion (0–10)*	3 (2–3.25)	4 (2–4.25)	4 (3–5.5)	5 (3–6.25)

Abbreviations: C‐0.3°C, mild core cooling of ∆‐0.3°C from baseline; C‐0.8°C, mild core cooling of ∆‐0.8°C from baseline; CS, Cold Skin/Shell; TN, thermoneutral.

* Indicates a significant effect (*p* < 0.05) using a Friedmans ANOVA where post hoc comparisons using Wilcoxon signed rank tests can be interpreted as: ^a^significantly different (*p* < 0.008) (corrected for multiple comparisons) from TN, ^b^significantly different from CS, ^c^significantly different from C‐0.3°C, ^d^significantly different from C‐0.8°C.

### 3.3 Cardiorespiratory responses

**FIGURE 2:**
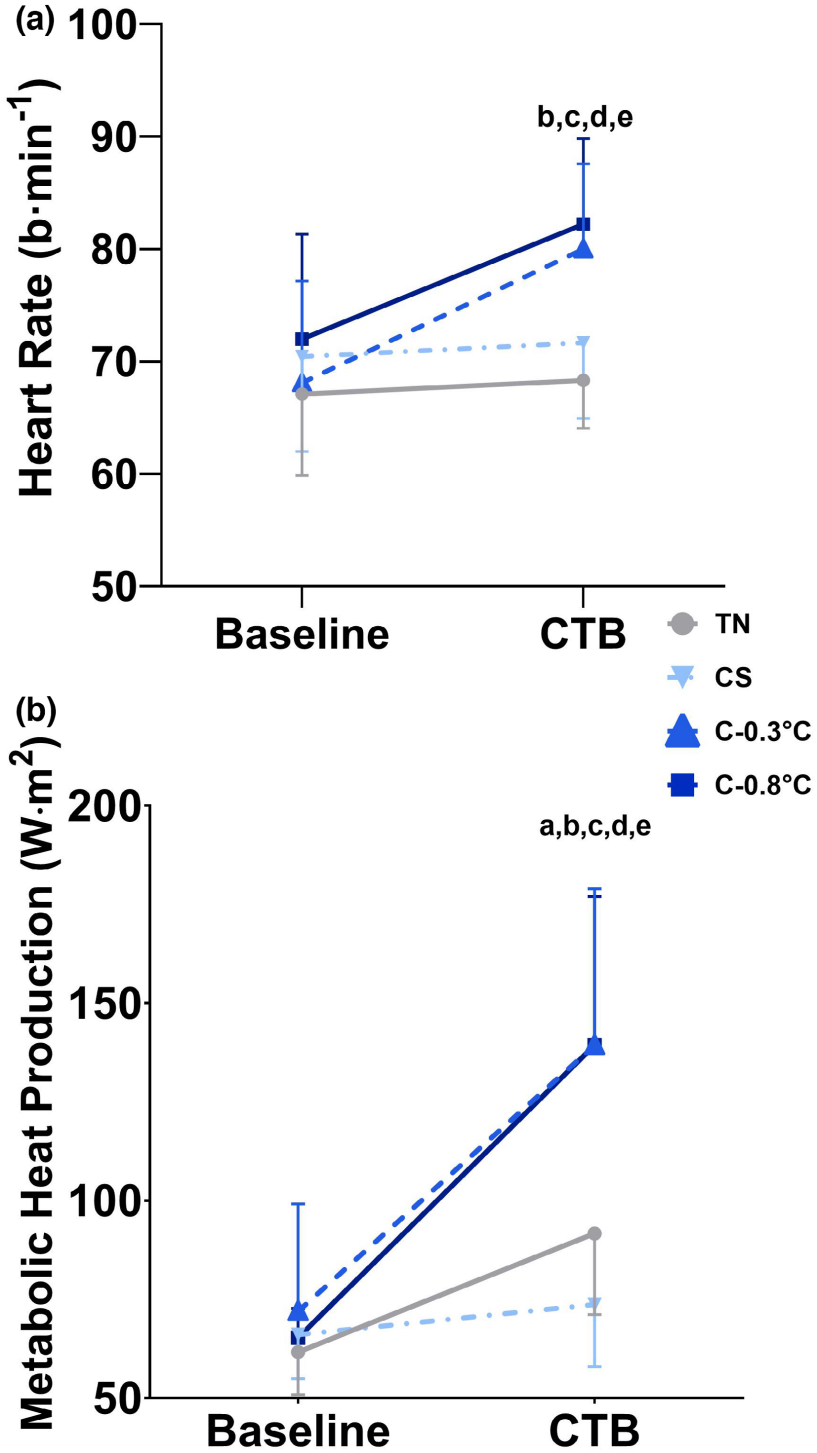
Heart rate (panel A) and metabolic heat production (panel B) responses (*n* = 10 males). Data were analyzed using a 2 Time × 4 Condition repeated measure ANOVA. All data are presented as mean ± SD. All data demonstrated a condition, time, and interaction effect (all *p* < 0.05). Pairwise comparisons can be interpreted as a = difference between TN and CS, b = difference between TN and C‐0.3°C, c = difference between TN and C‐0.8°C, d = difference between CS and C‐0.3°C, e = difference between CS and C‐0.8°C, f = difference between C‐0.3°C and C‐0.8°C. TN = thermoneutral, CS = Cold Skin/Shell, HYPO‐0.5°C = mild core cooling (hypothermia) of ∆‐0.5°C from baseline, HYPO‐1.0°C = mild core cooling (hypothermia) of ∆‐1.0°C from baseline.

There were effects for condition, time, and interaction (all *p* ≤ 0.03) for heart rate (Figure 2a) and $\dot{M}$ (Figure 2b) with no differences at Baseline (all *p* > 0.05). During the CTB, heart rate was higher in for both C‐0.3°C (*p* = 0.002) and C‐0.8°C (*p* = 0.003) compared to TN with no differences between the two conditions (*p* = 1.00). Additionally, heart rate was significantly higher in C‐0.3°C (*p* = 0.047) and C‐0.8°C than CS (*p* = 0.031). Metabolic heat production increased in all cold conditions; however, it was only significantly higher in both C‐0.3°C and C‐0.8°C compared to both TN and CS (all *p* ≤ 0.001), with no differences in $\dot{M}$ between C‐0.3°C and C‐0.8°C (*p* = 1.00), nor TN and CS (*p* = 0.141).

### 3.4 Cognitive performance

Cognitive performance for the simple reaction task, vertical flanker, and item working memory for all four experimental conditions are presented in Table 2. For simple reaction time, there was an effect for RSI (*p* ≤ 0.001, η_p_^2^ = 0.784), condition (*p* ≤ 0.001, η_p_^2^ = 0.467), but no interaction (*p* = 0.169, η_p_^2^ = 0.150). Pairwise comparisons demonstrated that RT was longer for 500 ms RSI (304 ± 40 ms) compared to 1000 ms RSI (274 ± 36 ms, *p* ≤ 0.001) and 1500 ms RSI (271 ± 36 ms, *p* ≤ 0.001). There was no difference between 1000 ms and 1500 ms RSIs (*p* = 1.000). Pairwise comparisons for the condition effect demonstrated slower RTs (~29 ms) in C‐0.3°C (*p* = 0.035) and C‐0.8°C (*p* = 0.008) compared to CS. There was no condition effect for a preparation effect (*p* = 0.088) or accuracy (*p* = 0.493) for simple reaction time.

**TABLE 2:** Cognitive performance responses (presented as mean ± SD) for the four experimental conditions (*n* = 10 males).

Variable	TN	CS	C‐0.3°C	C‐0.8°C
Simple reaction time task–all				
Reaction time (ms)* ^,^ ^†^	273 ± 38	267 ± 26^cd^	297 ± 33^b^	296 ± 41^b^
Accuracy (%)	98 ± 2	98 ± 2	97 ± 3	97 ± 4
Simple reaction time task–RSI				
500 RSI reaction time (ms)	292 ± 45	284 ± 24	323 ± 38	321 ± 47
1000 RSI reaction time (ms)	263 ± 36	258 ± 26	290 ± 35	283 ± 40
1500 RSI reaction time (ms)	264 ± 38	260 ± 60	277 ± 35	282 ± 41
Simple reaction time task–preparation effect				
Reaction time (ms)	−28 ± 22	−25 ± 16	−40 ± 20	−37 ± 25
Vertical Flanker–All				
Reaction time (ms)^†^	536 ± 67	527 ± 46	565 ± 54	573 ± 100
Errors (#)^†^	3 ± 3	3 ± 2	4 ± 3	3 ± 2
Accuracy (%)^†^	97 ± 4	96 ± 2	96 ± 3	96 ± 3
Vertical flanker–congruent stimuli				
Reaction time (ms)	504 ± 69	494 ± 47	530 ± 54	542 ± 94
Errors (#)	1 ± 1	1 ± 1	1 ± 1	0 ± 1
Accuracy (%)	98 ± 2	98 ± 2	97 ± 3	98 ± 3
Vertical flanker–incongruent stimuli				
Reaction time (ms)	568 ± 66	561 ± 47	601 ± 54	606 ± 107
Errors (#)	2 ± 3	2 ± 2	3 ± 3	3 ± 2
Accuracy (%)	96 ± 6	94 ± 3	94 ± 5	94 ± 3
Vertical flanker interference effect				
Reaction time (ms)	64 ± 20	66 ± 16	71 ± 21	60 ± 15
Item working memory–all				
Reaction time (ms)* ^,^ ^†^	844 ± 186	827 ± 198	922 ± 223	900 ± 235
Errors (#)^†^	4 ± 3	4 ± 2	4 ± 2	4 ± 3
Accuracy (%)^†^	87 ± 11	86 ± 8	88 ± 5	86 ± 10
Item working memory–two items				
Reaction time (ms)	673 ± 104	674 ± 115	762 ± 139	751 ± 210
Errors (#)	1.0 ± 1.2	1.0 ± 0.7	1.0 ± 1.0	1.0 ± 0.5
Accuracy (%)	93 ± 12	95 ± 7	93 ± 10	93 ± 13
Item working memory–four items				
Reaction time (ms)	843 ± 171	884 ± 219	904 ± 194	969 ± 305
Errors (#)	1 ± 1	1 ± 1	1 ± 1	1 ± 1
Accuracy (%)	89 ± 12	87 ± 12	93 ± 7	85 ± 11
Item working memory–six items				
Reaction time (ms)	987 ± 249	931 ± 297	1048 ± 346	947 ± 244
Errors (#)	2 ± 1	3 ± 1	2 ± 1	2 ± 2
Accuracy (%)	79 ± 15	74 ± 16	77 ± 12	80 ± 23

Abbreviations: C‐0.3°C, mild core cooling of ∆‐0.3°C from baseline; C‐0.8°C, mild core cooling of ∆‐0.8°C from baseline; CS, Cold Skin/Shell; TN, thermoneutral.

^†^ Indicates a significant response‐stimulus interval (RSI) effect for detection task or flanker effect for vertical flanker task or set‐size effect for item working memory task.

* Indicates a significant condition effect where pairwise comparisons can be interpreted as: ^a^significantly different from TN, ^b^significantly different from CS, ^c^significantly different from C‐0.3°C, ^d^significantly different from C‐0.8°C.

Vertical flanker RT demonstrated an effect for flanker type (*p* ≤ 0.001, η_p_^2^ = 0.969), however no effect for condition (*p* = 0.097, η_p_^2^ = 0.284) or interaction (*p* = 0.578, η_p_^2^ = 0.074). Pairwise comparisons revealed participants had a longer RT in incongruent trials (576 ± 54 ms) compared to congruent trials (511 ± 55 ms). For errors made, there was an effect for flanker type (*p* = 0.005, η_p_^2^ = 0.655), with no condition (*p* = 0.238, η_p_^2^ = 0.158) or interaction (*p* = 0.496, η_p_^2^ = 0.093). Pairwise comparisons revealed participants committed more errors on incongruent trials (2 ± 2) compared to congruent trials (1 ± 1, *p* = 0.005). For accuracy, there was an effect for congruency (*p* = 0.014, η_p_^2^ = 0.551) with no condition (*p* = 0.580, η_p_^2^ = 0.077), nor interaction (*p* = 0.708, η_p_^2^ = 0.055). Participants were more accurate in congruent trials (98 ± 2%) compared to incongruent trials (95 ± 4%, *p* = 0.014). There was no condition effect for the interference effect (all *p* > 0.05).

Item working RT had a significant effect for set size (*p* = 0.006, η_p_^2^ = 0.569) with no condition (*p* = 0.175, η_p_^2^ = 0.165) or interaction (*p* = 0.231, η_p_^2^ = 0.148). Overall, pairwise comparisons revealed the RT for 2 items (715 ± 148 ms) was significantly faster compared to 4 items (900 ± 234 ms, *p* = 0.016) and 6 items (976 ± 279 ms, *p* = 0.021). Four items RT were not significantly different from 6 items (*p* = 0.082). For errors made on the item working memory, there was a significant set‐size effect (*p* ≤ 0.0001, η_p_^2^ = 0.634), but no condition (*p* = 0.799, η_p_^2^ = 0.036) or interaction (*p* = 0.801, η_p_^2^ = 0.05). For accuracy, there was a significant set‐size effect (*p* ≤ 0.0001, η_p_^2^ = 0.613), but no condition (*p* = 0.893, η_p_^2^ = 0.022) or interaction (*p* = 0.670, η_p_^2^ = 0.070). Pairwise comparisons revealed participants committed more errors with reduced accuracy on six items (2 ± 2 errors, 78 ± 16%) compared to two items (1 ± 1 errors, *p* = 0.003, 94 ± 10%, *p* = 0.004) and four items (1 ± 1 errors, *p* = 0.028, 89 ± 11%, *p* = 0.033). When comparing two items compared to four items, both the # of errors made (*p* = 0.054) or accuracy (*p* = 0.113) were not different.

## 4 DISCUSSION

This study tested the effects of cold air exposure leading to skin and core temperature decreases on executive function, working memory and psychomotor processing. We hypothesized that cognitive performance would be impaired with decreases in skin temperature due to increased thermal discomfort (Teichner, 1958), with further reductions in performance with progressively greater core cooling (Ellis, 1982). We found that neither reductions in skin temperature, nor core temperature of ∆‐0.3°C and ∆‐0.8°C significantly impacted executive attention based cognitive process (i.e., temporal preparation effect (simple reaction time task), flanker effect (vertical flanker), set‐size effect (item working memory)). Furthermore, there was no significant slowing of RT, nor more errors made in any of the cold conditions compared to TN. Although there were slower RTs (~30 ms) on the simple reaction test in both core cooling conditions compared to CS however, from a practical standpoint, this magnitude of change would not be expected to affect overall acute cognitive performance for young healthy males. Combined, these data demonstrate that executive function, working memory, and psychomotor processing are generally well maintained during cold air exposure at magnitudes of up to ∆‐0.8°C core temperature decrease from baseline temperature.

While many studies report an impairment in cognitive function with cold air exposure, this finding is not universal. In the current study, the general pattern of response was a slowing of RT by ~24–78 ms in C‐0.3°C and ~24–56 ms in C‐0.8°C from TN depending on the task; however, we did not demonstrate any statistically significant changes in the two core cooling conditions compared to TN. Furthermore, we have no effect of cold on the number of errors made or accuracy on any task. We attempted to control for individual differences in thermoregulatory capacity by normalizing cold strain based on changes in skin temperature as well as normalizing the relative core temperature decrease from baseline as opposed to using a time‐based approach. One obvious explanation for the lack of impairment found in the present study could be that the level of core cooling was not sufficiently stressful thermally to impair cognitive performance. However, we are confident that in both core cooling conditions participants demonstrated significant relative cold strain as compared to baseline in thermoneutral conditions, as there were large decreases in ${\bar{T}}_{\text{skin}}$ (~∆‐9.5 to ‐11.4°C), moderate level of shivering (indicated by ≥~2x increase in $\dot{M}$ and higher heart rates by ~10–15 beats.min^−1^), and high perceptual thermal strain in the C‐0.3°C and C‐0.8°C conditions. Overall, these findings are in line with previous studies in cold air causing core cooling where decreases in core temperature did not impair executive function, working memory, or simple reaction time in cold air (−12 to 7.5°C) over 100 min to 24 h (Ellis, 1982; Færevik et al., 2021; Makinen et al., 2006; Taber et al., 2016). However, these are in contrast to impaired RT and increased errors made on a serial choice reaction time on a mathematical task (Ellis, 1982). Potentially, the null findings could be explained by the fact that the absolute core temperature (~36.5°C in C‐0.8°C) was not sufficient to impair cognitive function as no participants experienced clinical hypothermia (core temperature <35°C). Giesbrecht et al. (1993) determined that cold water immersion reducing esophageal core temperature to 33–35°C impaired executive function and working memory performance, but not simple task performance, suggesting that a lower absolute core temperature may be necessary for impairment. Future work is necessary to delineate core temperature thresholds more finely on impairments of cognitive functions; however, this may not be feasible in practice as ethical core temperature cutoffs are typically ≥35.0°C, nor practically useful from an operational or work performance standpoint.

A confounding variable for testing RT in the cold is the well‐documented decrease in manual dexterity (Castellani & Tipton, 2016), which can occur with both local temperature changes to forearm and hand temperature (Castellani et al., 2018; Giesbrecht et al., 1995; Sun et al., 2022) as well as with reductions in core temperature (Cheung et al., 2007; Giesbrecht et al., 1995). For computerized cognitive assessments, the hands and fingers are required to respond and therefore can directly influence RT and errors made. The large decreases in forearm and hand temperature noted in our study would likely impair manual dexterity, which we did not directly test but attempted to minimize. In pilot testing, we first attempted to normalize the manual dexterity requirements by having individuals wear the same winter gloves for testing while responding using a standard computer keyboard. However, participant feedback indicated that regardless of thermoneutral or cold conditions, participants perceived that false errors were occurring through missing the response buttons due to the bulkiness of the gloves, or in the cold condition due to cold hands and fingers. Therefore, we manipulated the keyboard to include two raised large analogue thumbsticks in order to create an easier platform for responses. This is an unvalidated tool and we did not control for participants using a single finger (e.g., second digit) or multiple fingers (e.g., second digit and third digit) to respond, but did instruct for their approach to be consistent during familiarization and experimental trials. This may have potentially contributed to the null effect in cognitive function, as the dexterity requirements were minimized. However, despite the altered manual dexterity requirement and environmental manipulations, each task were valid measures of the cognitive function tested as we demonstrated common task performance responses including the temporal preparation effect (simple reaction time), flanker effect (vertical flanker), and set‐size effect (item working memory) for RT, errors, and accuracy (Jones et al., 2015, 2016). Future studies in the cold may benefit from similar button configurations to minimize the dexterity requirements, however research is needed to see how different configurations affect RT and errors made. Furthermore, as manual dexterity is considered a major performance problem experienced in the cold (Castellani & Tipton, 2016), future studies should include information regarding hand conditions (e.g., wearing gloves, uncovered), local hand and forearm temperature, as well as method used to respond to cognitive tasks (e.g., keyboard, touch screen, button configuration). This approach to more carefully consider the various influencing factors may aid in clarifying confounding variables for the mixed results in cognitive function between studies.

Thermal displeasure from decreases in $\bar{T}$_skin_ has been shown to impair cognitive function before changes in core temperature through decreases in arousal and increases in distraction requiring multi‐tasking to focus on the task and monitor thermal state (Cheung et al., 2007; Teichner, 1958). Furthermore, it has been reported that cooling $\bar{T}$_skin_ to ~30°C can slow neuronal conduction velocity and central processing (Nakata et al., 2019). In the current study, we found that cooling $\bar{T}$_skin_ to ~27°C increased discomfort and perception of feeling cold; however, we found no differences for RT or errors made on any of the cognitive tasks when compared to TN. Overall, these results indicate that increased discomfort and distraction did not significantly influence cognitive performance relative to TN. We did see a significantly slower RT on the simple reaction time task (~30 ms) compared to both C‐0.3°C and C‐0.8°C compared to CS. From a real‐world application standpoint, the statistically significant changes in RT are not considered practically significant for relatively young healthy individuals working in environments where there is an opportunity to prepare (i.e., don weather appropriate clothing) in advance and prevent high degrees of shivering and mild core cooling. This suggestion is supported in that the changes found in this study were relatively small given the increased physiological and psychological strain in the core cooling conditions. Furthermore, there was a significant level of whole‐body shivering (Figure 2b) and local cooling of the forearm and hand, which may have increased the motor demands, influenced coordination for responding, or influenced manual dexterity leading to small differences in RT. Most importantly, these changes did not influence the number of errors made or accuracy and did not extend to more complex vertical flanker and item working memory tasks. However, given the changes that were identified, future research is needed to better isolate and identify the specific $\bar{T}$_skin_ or *T*_core_ threshold at which impairment begins on RT. Furthermore, future studies would benefit from determining individualized responders in impairment in cognitive function under cold stress.

A strength of the research design was controlling for both changes in skin temperature as well as relative changes in core temperature to determine the separate and combined effects of cold on cognitive function. This approach took into account the individual variability of cooling (due to anthropometry, shivering ability, vasoconstriction) and normalized physiological cold strain. However, this approach leads to differences in the rate of cooling between participants, where future research is needed to determine if the overall rate of skin and/or core cooling influences cognitive function. Typically, studies testing cognition and cold stress have sample sizes ranging from 6 to 12 participants due to high thermal strain and logistical challenges (Ellis, 1982; Færevik et al., 2021; Gibbons et al., 2020; Jones et al., 2019; Mahoney et al., 2007; Makinen et al., 2006; Muller et al., 2012; Shurtleff et al., 1994; Taber et al., 2016; Thomas et al., 1989). We were sufficiently powered for measures to test cognitive function such as the RT flanker effect for the vertical flanker task (power analysis estimated *n* = 8 required to achieve α = 0.05 and ß = 0.8), however, we were insufficiently powered for the condition effect (power analysis estimated *n* = 28 required) due to low effect size and individual variability in performance. A limitation of the current study is that the task complexity may not have been high enough to induce impairments, as the median ratings of perceived mental exertion was 3–5 out of 10 (“moderate” to “hard”), and participants may have retained sufficient neural resources to complete the tasks. Previous studies have found impairment in working memory with increased task complexity (Mahoney et al., 2007; Shurtleff et al., 1994). We demonstrated that individuals made more errors and were slower to respond as the set number increased from two to six items, however we found no impairments in performance collectively or at each level of difficulty due to cold. Furthermore, we cannot account for any central changes in neural function including cerebral blood flow (Gibbons et al., 2020) or electrical activity (using electroencephalography) (Jones et al., 2019) limiting our understanding of neural changes during cognitive tasks. Recently, Jones et al. (2019) determined that core cooling by ~1.5°C with cold water immersion increased the requirement for pre‐attention (N100) and processing effort (P300) using electroencephalography on a psychomotor vigilance task, indicating higher cognitive load with mild hypothermia. Previously, Qian et al. (Qian et al., 2015) found that passive heat stress increases the onset of mental fatigue, and currently it is unknown if the increased cognitive load leads to a faster onset of mental fatigue in the cold. Lastly, there are circadian fluctuations in core body temperature (e.g., core temperature lowers during the nighttime) (Færevik et al., 2021) that exist within the absolute core temperature ranges in the current study. However, we are confident that our participants experienced a sufficient core change greater than circadian rhythm changes, as in conjunction with core temperature changes, there were large decreases in whole‐body skin temperature, local forearm and hand temperature (for task responses), thermal sensations and increased levels of shivering, heart rate, and thermal discomfort indicating significant cold strain.

In summary, we demonstrated that a decrease in both skin and core temperature combined with increased perceptual thermal strain and shivering after cold air exposure did not impair executive function, working memory or psychomotor processing compared to thermoneutral conditions as indexed by maintenance of cognitive processes and no changes in errors made. There is evidence for a slowing of reaction time, however this was not significantly different from TN in any cold condition. Future research is needed to determine the threshold for impairment in these functions as well as determining task dependent changes that occur in cold air environments. Furthermore, future research is needed to determine how longer exposures and/or different modes of cold stress may affect cognition.

## AUTHOR CONTRIBUTIONS

All authors contributed to the conception and design of the research study; Phillip J. Wallace piloted and performed the experiments and the statistical analysis. All authors interpreted the results of the study. Phillip J. Wallace, Michael J. Taber, and Stephen S. Cheung drafted the manuscript. All authors edited, revised, and approved the final version of the manuscript.

## FUNDING INFORMATION

This study was supported by a Discovery grant from the Natural Science and Engineering Research Council (NSERC) of Canada (SSC, 2018‐04077). PJW was supported through a NSERC Doctoral (PGS D) scholarship, Ontario Graduate Scholarship, and Queen Elizabeth II Graduate Scholarship in Science & Technology over the course of this research.

## CONFLICT OF INTEREST STATEMENT

The authors declare that the research was conducted in the absence of any commercial or financial relationships that could be construed as a potential conflict of interest. The data that support the findings of this study are available from the corresponding author upon reasonable request.

## ETHICS STATEMENT

Each participant provided both verbal and written informed consent that was approved by the Research Ethics Board at Brock University (REB#19‐026).
